# Caloric restriction exerts site-, sex-, and duration-dependent effects on skeletal structure and bone marrow adiposity

**DOI:** 10.1530/JOE-25-0391

**Published:** 2026-05-11

**Authors:** Kuan-Chan Chen, Richard J Sulston, Karla J Suchacki, Yoshiko M Ikushima, Benjamin J Thomas, Andrea Lovdel, Alex J Lafond, Sharon E Mitchell, John R Speakman, Nicholas M Morton, Robert K Semple, William P Cawthorn

**Affiliations:** ^1^ Institute for Neuroscience and Cardiovascular Research, The University of Edinburgh, Edinburgh BioQuarter, Edinburgh, UK; ^2^ Division of Endocrinology and Metabolism, Department of Internal Medicine, Tri-Service General Hospital, National Defense Medical University, Taipei, Taiwan; ^3^ Scotland’s Rural College, The Roslin Institute, Edinburgh, UK; ^4^ Department of Medical Science and Innovation, SiRIUS Institute of Medical Research, Tohoku University, Sendai City, Miyagi, Japan; ^5^ Institute of Biological and Environmental Sciences, University of Aberdeen, Aberdeen, UK; ^6^ Centre for Systems Health and Integrated Metabolic Research, Department of Biosciences, School of Science and Technology, Nottingham Trent University, Nottingham, UK

**Keywords:** caloric restriction, bone marrow adipose tissue, bone microarchitecture, trabecular bone, cortical bone, energy metabolism, endocrinology, sex differences

## Abstract

Bone marrow adipose tissue (BMAT) is a unique fat depot. It has distinct metabolic–endocrine functions and negatively correlates with bone mineral density, increasing with ageing and osteoporosis. Intriguingly, BMAT also expands during caloric restriction (CR), a lifespan-extending dietary intervention that also promotes bone loss; however, whether BMAT is a cause or consequence of this remains unclear. To address this, we studied 9-week-old male and female C57BL/6NCrl mice subjected to 30% CR for 1, 2, 4, or 6 weeks. Bone structure and BMAT volume were quantified by micro-computed tomography using a spatial method that precisely maps BMAT within bones. CR-induced BMAT expansion was site-specific, being greater in tibiae than femora and absent in humeri. Surprisingly, CR increased distal tibial BMAT despite prior suggestions that this BMAT region resists such environmental modulation. Expansion was also duration-dependent, plateauing after 4 weeks, and region-specific, particularly in the tibial metaphysis, femoral metaphysis, and the distal tibia. Similarly, CR altered trabecular and cortical bone in time-, region-, and sex-dependent ways, with BMAT levels more tightly associated with trabecular than cortical changes. CR also increased adiponectin, corticosterone, and ketones, while decreasing leptin, IGF-1, and insulin, in a sex- and/or duration-dependent manner. Metabolic phenotyping demonstrated that BMAT expansion was not associated with altered glucose tolerance, body mass, total fat mass, or fat percentage but correlated with energy deficit and systemic lipid mobilisation. Together, our findings highlight the complex interplay between BMAT expansion, skeletal remodelling, and metabolic homeostasis, providing new insights into BMAT formation and function and the health benefits of CR.

## Introduction

Bone marrow adipose tissue (BMAT) is a distinct fat depot comprising up to 70% of bone marrow volume and >10% of total adipose mass in healthy adults (1, 2). BMAT secretes adipokines such as adiponectin, thereby potentially influencing systemic metabolism, bone remodelling, and haematopoiesis (3). There are two major BMAT subtypes: regulated BMAT (rBMAT), which is interspersed with haematopoietic tissue, and constitutive BMAT (cBMAT), which is morphologically similar to white adipose tissue, more stable than rBMAT, and enriched in distal skeletal sites (4). This heterogeneity may contribute to BMAT’s region-specific skeletal and metabolic functions.

BMAT correlates inversely with bone mineral density (BMD) (5) and frequently increases in osteoporotic states, including ageing, menopause (6), glucocorticoid excess (7, 8), and anorexia nervosa (9). Notably, recent UK Biobank analyses from us and others have identified direct causal associations between BMAT and osteoporosis (10, 11, 12).

Unlike other adipose depots, BMAT also increases during caloric restriction (CR) (2, 13), a dietary intervention that promotes longevity and metabolic health across multiple species (14, 15, 16). Despite these health benefits, CR is frequently associated with reduced bone mass (17). These contrasting effects raise fundamental questions about the role of BMAT during CR: does BMAT expansion exert metabolic benefits, or might it also contribute to CR-induced skeletal fragility?

Most studies of CR-induced BMAT expansion have focused on single time points, isolated skeletal regions, and only one sex, underscoring the need for further investigation into the dynamic, spatial, and sex-specific features of this phenomenon (13, 18, 19, 20). To address these gaps, we conducted a comprehensive, time-resolved analysis of BMAT and bone structure in male and female mice across multiple CR durations, analysing diverse skeletal sites using micro-computed tomography (μCT) with and without osmium tetroxide staining. We also analysed the relationships between BMAT expansion and the metabolic and endocrine effects of CR. This design enabled us to define the anatomical and temporal features of BMAT expansion; assess its relationship with bone remodelling, metabolic and endocrine adaptations; and determine sex-specific responses. Together, our findings provide new insights into BMAT’s fundamental physiological functions and contributions to CR’s impact on skeletal and metabolic health.

## Materials and methods

### Animals and CR

All animal procedures were approved by the University of Edinburgh Animal Welfare and Ethical Review Board or the University of Aberdeen ethical approval committee and conducted under UK Home Office project licenses.

C57BL/6NCrl mice (Charles River, UK) were bred and maintained in-house under specific pathogen-free conditions on a 12 h light:12 h darkness cycle. The CR protocol is as described previously (21). Briefly, CR mice received 70% of the average daily AL intake, provided daily during 09:00–10:00. CR was maintained for 1, 2, 4, or 6 weeks, depending on the experimental cohort. Study design and randomisation were done as described previously (21). Sample size estimation was based on the primary outcome of CR-induced tibial BMAT expansion, with power calculations (G*Power software) using effect sizes observed in our previous studies (3). Because mice were singly housed, an individual mouse was used as the experimental unit of analysis. Nine mice were excluded because of a technical issue during gavage for oral glucose tolerance tests, which compromised their subsequent food intake. Several 1-week AL and CR female tibiae were also excluded from the analyses because of technical issues that invalidated the μCT data. Exact numbers of mice used are stated in the figure legends and the source data.

This CR regimen was used for all mice except for those undergoing graded CR (used for additional analysis of humerus BMAT). These mice underwent 0, 10, 20, 30, or 40% CR from 20 to 32 weeks of age as described (22).

### Bone analysis by μCT and BMAT quantification

Analysis of bone microarchitecture and BMAT by μCT was done as described previously (23). All scans were reconstructed using NRecon software (Bruker, Belgium). Bone length was determined from μCT datasets by multiplying the number of slices between the proximal and distal ends by the slice thickness (voxel size, μm).

To account for variability in bone length resulting from different durations of CR (1, 2, 4, or 6 weeks), each bone was digitally segmented into 100 equal-length percentile intervals along its longitudinal axis. Tibial cortical bone and BMAT analyses were conducted within the region spanning 10–80% of the bone length, while femoral cortical bone analyses were limited to the 25–85% region to minimise interference from the proximal and distal metaphyseal areas; for these regional analyses, cortical parameters were calculated using 2D analyses because each sub-region accounts for only a small volume along the bone length. For the humerus cortical bone, analyses focused on the 30–40% region of the bone and cortical parameters were calculated using 3D analyses because they correspond to a larger single bone volume. For trabecular bone, a volume of interest (VOI) was defined in the proximal tibial or distal femoral metaphysis, extending 5% of the total tibial length from the bottom of the growth plate, excluding the cortical shell.

Image analysis was done in CTAn software (Bruker) using automated region of interest (ROI) selection according to the manufacturer’s instructions. All ROIs were subsequently reviewed and manually refined to ensure accurate anatomical segmentation. BMAT was quantified as the ratio of osmium-stained adipose volume (Ad.V, from osmium-stained scans) to medullary cavity volume (Ma.V, from undecalcified scans) within the same anatomical region (BMAT% = Ad.V/Ma.V × 100), as recommended by guidelines from the International Bone Marrow Adiposity Society (1). The exception is for humeral BMAT from graded CR mice (Supplementary Fig. 3 (see section on Supplementary materials given at the end of the article)), which are reported as absolute BMAT volume (mm^3^) because bones were scanned only after decalcification and osmium staining.

### Histology

Vertebrae were decalcified by incubating in 14% EDTA solution at 4°C for 14 days, with the EDTA solution replaced every 3 days. Decalcified vertebrae were fixed, paraffin-embedded, sectioned, and stained with haematoxylin and eosin (H&E). Adipocyte size distribution was quantified as described previously (21).

### Metabolic and endocrine analyses

Oral glucose tolerance tests (OGTTs) were performed on male and female AL and CR mice at 1, 2, 4, and 6 weeks of dietary intervention, as described previously (21).

Plasma hormones were measured with commercial assay kits using the manufacturer’s protocols: adiponectin (MRP300, Bio-Techne, USA), leptin (MOB00B, Bio-Techne, USA), IGF-1 (ab100695, Abcam, UK), insulin (Crystal Chem, 90080, Crystal Chem, UK), and corticosterone (ADI-900-097, Enzo Life Sciences, USA). Blood ketones were measured using a GKI-Bluetooth Blood Glucose & Ketone Meter (Keto-Mojo Europe, Amsterdam, Netherlands). Adiponectin and ketone data were reported previously (21, 24) and are here compared to BMAT for the first time.

Body composition and indirect calorimetry data, including energy expenditure and respiratory exchange ratio (RER), were measured as described previously (21). Fatty acid oxidation was determined as described (25). *De novo* lipogenesis was approximated from the area under the curve (AUC) for RER values > 1. Herein, these previously published datasets were reanalysed to assess their associations with BMAT volume.

### Statistical analyses

Data were analysed using GraphPad Prism (v10.4.2) and R (v4.4.1). Two-way or three-way ANOVA was used to assess effects and interactions of sex, diet, duration, and/or bone location, with Sidak’s multiple comparison tests applied where appropriate.

For spatial analyses within an individual bone, three-way ANOVA was performed to compare outcomes across anatomical locations. Node-wise *P*-value heatmaps were generated to visualise region-specific differences along the tibiae and femora. Following two-way ANOVA, Fisher’s least significant difference (LSD) test was used to assess the pairwise difference between sexes or diets at individual bone segments. *P* values were not adjusted across segments because of the strong spatial correlation between adjacent locations, meaning that the node-wide tests are not independent. Consequently, applying strict multiple-testing correction across all nodes (e.g. Bonferroni/Holm assuming independence) would be overly conservative and markedly inflate type II error, potentially obscuring biologically meaningful spatial patterns along the bone (at the cost of inflating type I error).

Simple linear regression was used to evaluate associations between BMAT (exposure) and trabecular or cortical bone parameters (outcomes) in the CR groups, or between metabolic exposures (energy balance, RER, fatty acid oxidation, or lipogenesis) and tibial BMAT (outcome). For the BMAT vs bone parameter analyses, effect sizes (beta coefficients) are based on the absolute or relative change in the outcome variable per 1% change in BMAT, with absolute changes corresponding to the units of measurement for each outcome variable (e.g. g/cm^3^ for BMD) and the relative changes corresponding to the percentage change in the outcome (normalised to the group mean for each outcome).

### Data availability

Source data are available on the Open Science Framework (https://doi.org/10.17605/OSF.IO/CDNU8).

## Results

### CR induces duration-dependent BMAT expansion in the tibia

To determine if CR-induced BMAT expansion depends on CR duration, we fed 9-week-old male and female C57BL/6NCrl mice AL or a CR diet (70% of daily AL intake) for one, two, four, or six weeks. We then quantified BMAT by μCT of osmium tetroxide-stained bones, beginning with tibiae because they have been well characterised in previous CR studies (3, 18, 19, 23).

**Figure 1:**
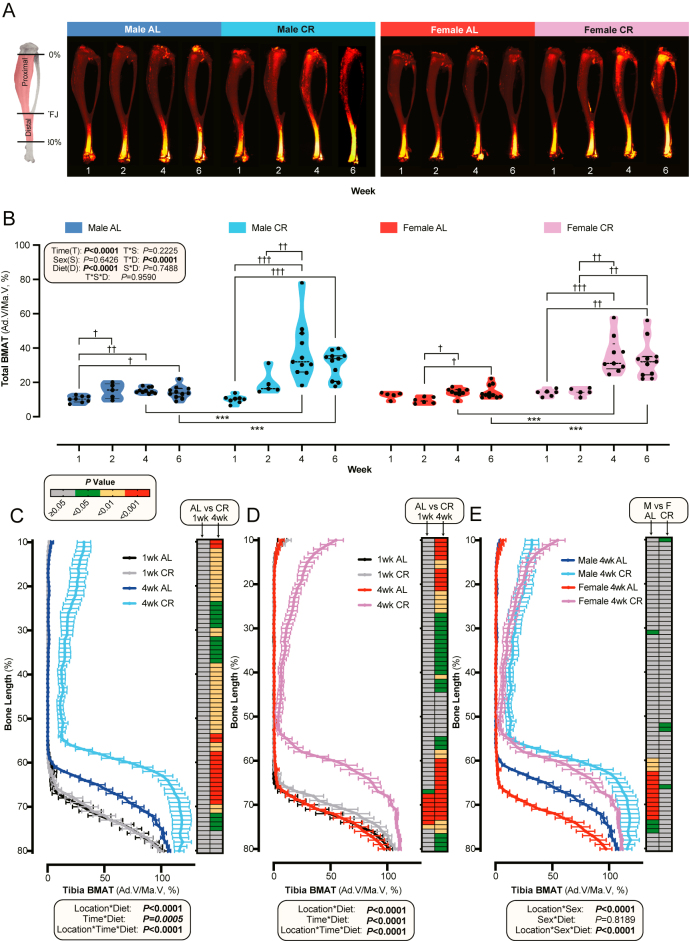
Impact of CR on BMAT in the tibia. Male and female mice were fed either an AL or a 30% CR diet starting at 9–10 weeks of age for 1, 2, 4, or 6 weeks. (A) Representative osmium-stained μCT images of tibiae across all time points, diets (AL and CR), and sexes; TFJ = tibia-fibula junction. (B) Quantification of whole tibial BMAT (%) across time points, diets, and sexes. (C and D) BMAT (%) comparison between AL and CR groups at weeks 1 and 4 in males (C) and females (D). (E) BMAT (%) comparison across diets and sexes at week 4. Data are shown as violin plots (B) or mean ± SEM (C, D, E). The following numbers of mice per group: male AL, 1 week: *n* = 8, 2 weeks: *n* = 5, 4 weeks: *n* = 10, 6 weeks: *n* = 11; male CR, 1 week: *n* = 8, 2 weeks: *n* = 5, 4 weeks: *n* = 11, 6 weeks: *n* = 11; female AL, 1 week: *n* = 5, 2 weeks: *n* = 6, 4 weeks: *n* = 9, 6 weeks: *n* = 11; female CR, 1 week: *n* = 6, 2 weeks: *n* = 5, 4 weeks: *n* = 9, 6 weeks: *n* = 11. (B, C, D, E) Significant effects of diet, time, sex, and/or location, as well as their interactions, were assessed using three-way ANOVA. Two-way ANOVA and Post hoc Šidák’s tests identified (B) (1) differences between AL and CR at each time point, and (2) differences among time points within each diet group. Significance is indicated by *P < 0.05, **P < 0.01, ***P < 0.001 (AL vs CR), and ^†^*P* < 0.05, ^††^*P* < 0.01, ^†††^*P* < 0.001 (within-group time point comparisons). (C, D, E) For heatmap analysis, two-way ANOVA with Fisher’s LSD post hoc test was used to compare the effects of diet (AL vs CR) and sex (male vs female). Statistical significance is colour-coded as shown in the legend: red for *P* < 0.001, yellow for *P* < 0.01, green for *P* < 0.05, and grey for *P* ≥ 0.05.

Total tibial BMAT increased slightly with increasing age in AL mice and more markedly with increasing CR duration (Fig. 1A and B: significant time and time–diet interactions). After four weeks of CR, BMAT in both sexes increased approximately 2.5-fold relative to time-matched AL controls, with no further increase occurring from 4 to 6 weeks (Fig. 1B). Similar duration-dependent effects of CR were observed for separate analyses of the proximal and distal tibia (Supplementary Fig. 1A and B), corresponding to rBMAT and cBMAT, respectively, with BMAT reaching around 12-fold (rBMAT) and 1.4-fold (cBMAT) of AL levels at six weeks of CR in both sexes. There were no significant sex–diet interactions for total, proximal, or distal BMAT (Fig. 1B, Supplementary Fig. 1A and B), suggesting no global sex differences in CR-induced BMAT expansion. Together, these findings show that CR duration is a key determinant of tibial BMAT accumulation.

To further understand CR’s region-specific effects on tibial BMAT, we next analysed the spatial distribution of BMAT along the tibia length (Fig. 1C, D, E, Supplementary Fig. 1D and E). Here, BMAT and bone parameters could be compared for each slice along the bone length, as shown by the *P* value heatmaps alongside each graph. Because tibial length varied (Supplementary Fig. 1C), BMAT distribution was analysed according to relative tibial length to allow direct comparison of time and diet effects. Moreover, we focused our AL vs CR comparisons on the one-week and four-week groups because significant CR effects occurred after four weeks.

This new method revealed that CR’s duration-dependent effects on BMAT expansion differ by tibial location. First, BMAT expansion predominates in the proximal and distal tibia, rather than the central diaphysis (Fig. 1C, D, E). Second, CR significantly increased distal BMAT during the first week only in females (Fig. 1D; 1 week heatmaps), suggesting greater sensitivity than males to CR’s early effects on BMAT. Notably, when comparing 4-week AL and CR groups, males had more BMAT than females at the distal tibia under the AL diet, whereas under CR, females tended to have greater BMAT accumulation at the most-proximal tibial sites (Fig. 1E).

Comparison of BMAT across all CR durations revealed location-specific increases, in both sexes, between 1 vs 2, 1 vs 4, and 1 vs 6 weeks of CR (Supplementary Fig. 1D and E); however, BMAT tended to decrease between weeks 4 and 6, with distal BMAT decreasing significantly in females (Supplementary Fig. 1D and E). Thus, the effect of CR on tibial BMAT is duration-dependent and region-specific, with spatial distribution analyses revealing sex-specific patterns that are undetected by conventional techniques that measure total BMAT volume.

### CR induces duration-dependent tibial metaphyseal BMAT expansion and decreases trabecular thickness in females

**Figure 2:**
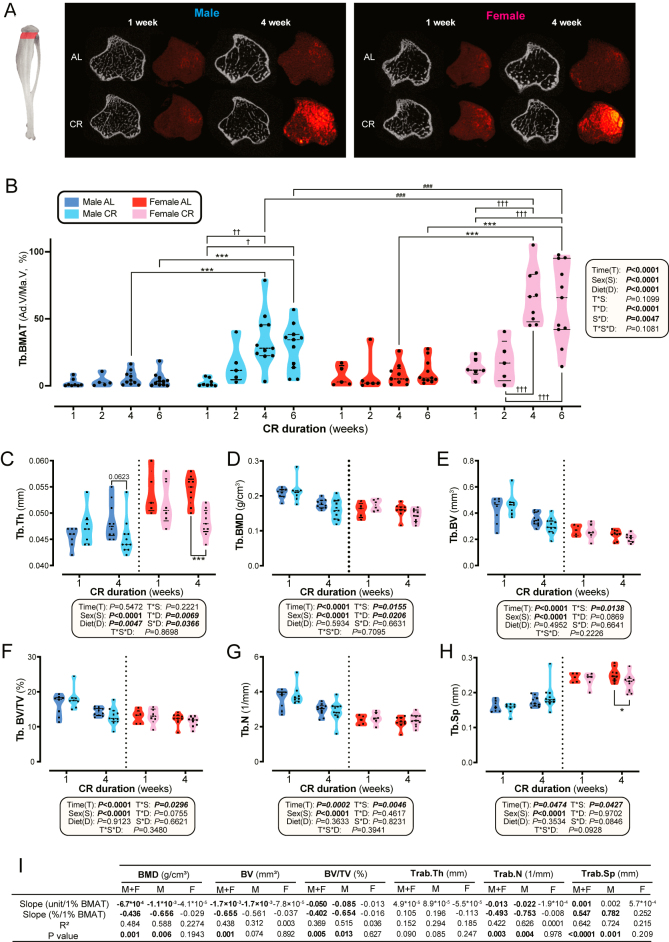
Impact of CR on trabecular bone microarchitecture in the tibia. Male and female mice were fed AL or 30% CR diets as described for Fig. 1. (A) Representative cross-sectional images of tibial bone microarchitecture and osmium-stained BMAT from μCT scans after 1 and 4 weeks of AL or CR feeding in male and female mice. (B) Quantification of tibial metaphyseal BMAT (%) across all time points, diet groups, and sexes. (C, D, E, F, G, H) Comparisons of tibia trabecular bone parameters between AL and CR groups in both sexes at week 1 and week 4, including (C) Tb.Th (D) Tb.BMD, (E) Tb.BV, (F) Tb.BV/TV, (G) Tb.N, and (H) Tb.Sp. Data in (B, C, D, E, F, G, H) are shown as violin plots. (I) Simple linear regression was used to evaluate associations between trabecular bone parameters and BMAT in the 4-week CR groups. Effect sizes are based on the absolute (unit) or relative (%) change in each bone parameter per 1% increase in BMAT, as explained in the Methods section. *R*^2^ and *P*-values are also reported for each regression. Sample sizes for (A, B, C, D, E, F, G, H, I) are as described for Fig. 1. In (B, C, D, E, F, G, H), significant effects of diet, time, and sex, as well as their interactions, were assessed using 3-way ANOVA. Two-way ANOVA and Post hoc Šidák’s tests identified differences between AL and CR at each time point. Significance is indicated by *P < 0.05, **P < 0.01, ***P < 0.001 (AL vs CR); ^†^*P* < 0.05, ^††^*P* < 0.01, ^†††^*P* < 0.001 (time point comparisons within CR group); ^#^*P* < 0.05, ^##^*P* < 0.01, ^###^*P* < 0.001 (male vs female comparisons within CR group).

We next investigated if the effects of CR on trabecular BMAT and bone microarchitecture are also duration-dependent. CR caused duration-dependent increases in tibial metaphyseal BMAT, but, unlike for whole tibiae, this expansion differed between the sexes, with metaphyseal BMAT increasing by approximately 6.1-fold in males and 7.9-fold in females after 4 weeks of CR (Fig. 2A and B).

Because metaphyseal BMAT peaked after 4 weeks, we compared trabecular parameters between 1-week and 4-week mice to assess duration dependence. When assessed across both sexes and time points, CR alone decreased trabecular thickness (Tb.Th), particularly in females, without altering any other trabecular indices (Fig. 2C, D, E, F, G, H). However, significant time–diet interactions occurred for Tb.Th and trabecular BMD (Tb.BMD), with prolonged CR progressively decreasing Tb.Th compared with AL mice (Fig. 2C and D). In contrast, no significant time–diet interactions occurred for trabecular bone volume (Tb.BV), bone fraction (Tb.BV/TV), number (Tb.N), or separation (Tb.Sp) (Fig. 2E, F, G, H). Notably, CR exerted sex-dependent effects on Tb.Th, decreasing this more in females than in males (Fig. 2C). This suggests that CR has stronger effects on bone microstructure in females, particularly after prolonged CR.

We next used simple linear regression to determine if these changes in trabecular parameters are related to BMAT expansion after 4-week CR. BMAT correlated negatively with Tb.BMD, Tb.BV, Tb.BV/TV, and Tb.N, and positively with Tb.Sp (Fig. 2I). Interestingly, although CR reduced Tb.BMD and Tb.Th in a duration-dependent manner (Fig. 2C and D), only Tb.BMD showed significant negative correlations with BMAT in CR mice (Fig. 2I, Supplementary Fig. 2A). Furthermore, CR did not affect Tb.BV, Tb.BV/TV, Tb.N, or Tb.Sp (Fig. 2E, F, G, H), yet these parameters showed significant relationships with BMAT during CR, particularly in males (Fig. 2I, Supplementary Fig. 2A). This supports the possibility that BMAT accumulation predicts a worsened trabecular bone microarchitecture during CR.

### CR induces duration-, sex-, and region-dependent reductions in tibial cortical bone

**Figure 3:**
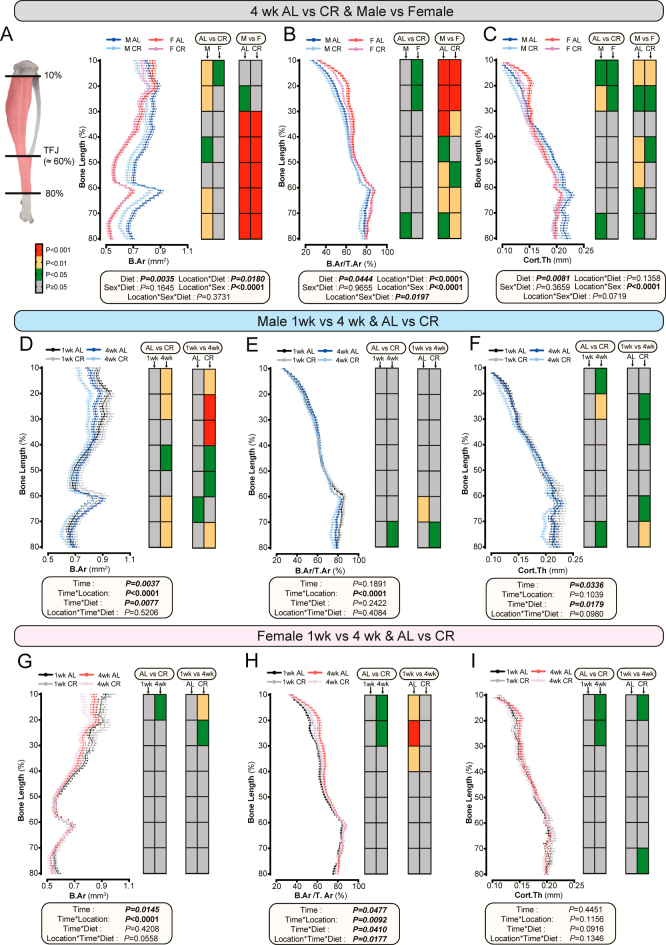
Impact of CR on cortical bone microarchitecture in the tibia. Male and female mice were fed AL or 30% CR diets as described for Fig. 1. (A, B, C) Comparisons of tibia cortical bone parameters between AL and CR groups after 4 weeks of AL or CR feeding, across 10–80% of tibial length in both sexes; TFJ = tibia-fibula junction. (A) B.Ar, (B) B.Ar/T.Ar, and (C) Cort.Th. (D, E, F, G, H, I) Comparisons of cortical bone parameters between 1-week and 4-week AL and CR groups in males (D, E, F) and females (G, H, I) across 10–80% of tibial length: (D and G) B.Ar, (E and H) B.Ar/T.Ar, and (F and I) Cort.Th. Data are shown as mean ± SEM (A, B, C, D, E, F, G, H, I). Sample sizes and heatmap analysis follow those described in Fig. 1 (A, B, C, D, E, F, G, H, I). Significant effects of diet, time, sex, and/or location, as well as their interactions, were assessed using three-way ANOVA. Two-way ANOVA with Fisher’s LSD post hoc test was used to compare the effects of diet (AL vs CR) and sex (male vs female). Statistical significance is colour-coded as shown in the legend: red for *P* < 0.001, yellow for *P* < 0.01, green for *P* < 0.05, and grey for *P* ≥ 0.05.

In rodents, CR-induced bone loss is greater for cortical vs trabecular bone (3, 23, 26). Thus, we next assessed if this effect is also duration- and/or sex-dependent. Comparing 4-week AL and CR groups in both sexes revealed that CR overall reduced cortical bone area (B.Ar), bone area fraction (B.Ar/T.Ar), and thickness (Cort.Th) and that these effects were location-dependent, differing along the length of the bone (Fig. 3A, B, C: ‘AL vs CR’ heatmaps). Sex differences were observed for all parameters; however, the spatial patterns of these differences varied across tibial locations (Fig. 3A, B, C: ‘M vs F’ heatmaps). Although no significant sex–diet interactions occurred at the whole-bone level, a significant location–sex–diet interaction occurred for B.Ar/T.Ar but not for B.Ar or Cort.Th (Fig. 3A, B, C). Therefore, sex does not modify CR’s overall effects on cortical bone but does influence the region-specific impact of CR on certain cortical parameters.

To further explore the temporal dynamics, we compared cortical parameters across 1-week and 4-week AL and CR mice (Fig. 3D, E, F, G, H, I). In either sex, CR for 1 week did not affect any parameter at any location along the bone length (Fig. 3D, E, F, G, H, I; ‘AL vs CR’ heatmaps); however, location-specific CR effects were apparent after four weeks. In males, the strongest time×diet effects occurred for B.Ar, which was significantly decreased after 4 weeks of CR (Fig. 3D). In contrast, time×diet effects in females were weaker, with CR suppressing the increase in B.Ar/T.Ar that occurred between weeks 1 and 4 in AL mice (Fig. 3H). Together, these data show that CR modulates cortical bone in a time-, sex-, and region-specific manner.

We next used simple linear regression to further assess the relationships between BMAT and cortical bone for 4-week CR mice, both across whole bones and within each decile along the bone length (Supplementary Fig. 2B). BMAT was negatively associated with Cort.Th in the mid-diaphyseal region of CR males, but no other significant relationships were detected. Thus, CR-induced BMAT expansion has only localised, limited relationships with cortical bone parameters, contrasting with its more pronounced, widespread relationships with trabecular bone (Fig. 2I, Supplementary 2A).

### CR induces duration-dependent femoral metaphyseal BMAT expansion with female-specific reductions in trabecular thickness

We next investigated if CR exerts duration-dependent effects on BMAT and bone microarchitecture in the femur, for which BMAT expansion has also been reported during CR (13). Even after six weeks of CR, femoral BMAT expansion predominantly occurred in the epiphysis and metaphysis, rather than in the diaphysis (data not shown). Therefore, we focused on the femoral metaphysis.

**Figure 4:**
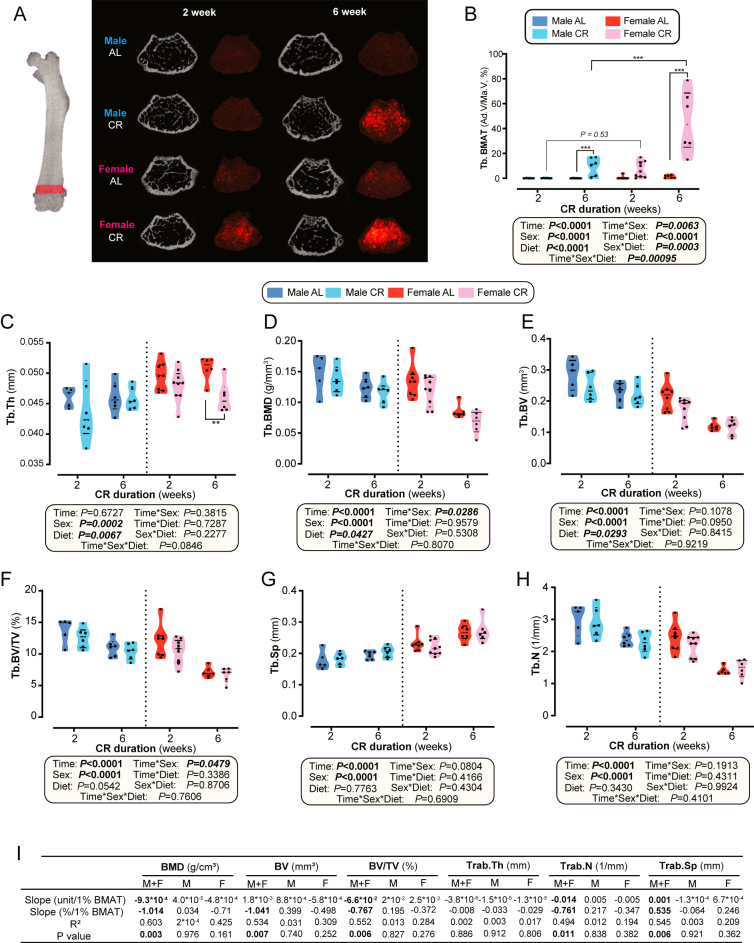
Impact of CR on trabecular bone microarchitecture in the femur. Male and female mice were fed AL or 30% CR diets as described for Fig. 1. (A) Representative cross-sectional images of femoral bone microarchitecture and osmium-stained BMAT from μCT scans after 2 and 6 weeks of AL or CR feeding in male and female mice. (B) Quantification of femoral metaphyseal BMAT (%) across diet and sexes in week 2 and week 6. (C, D, E, F, G, H) Comparisons of femoral trabecular bone parameters between AL and CR groups in both sexes at week 2 and week 6, including (C) Tb.Th, (D) Tb.BMD, (E) Tb.BV, (F) Tb.BV/TV, (G) Tb.Sp, and (H) Tb.N. Data in (B, C, D, E, F, G, H) are shown as violin plots. (I) Simple linear regression was used to evaluate associations between femoral trabecular bone parameters and BMAT in the 6-week CR groups; regression was done as described in Fig. 2I. Data in A, B, C, D, E, F, G, H, and I are from analysis of the following numbers of mice per group: male AL, 2 weeks: *n* = 5, 6 weeks: *n* = 6; male CR, 2 weeks: *n* = 6, 6 weeks: *n* = 6; female AL, 2 weeks: *n* = 8, 6 weeks: *n* = 6; and female CR, 2 weeks: *n* = 9, 6 weeks: *n* = 6. Statistical analyses follow the methods detailed in Fig. 2.

Similar to the tibiae, CR caused duration-and sex-specific increases in femoral BMAT: longer durations caused greater accumulation, and although no sex difference was observed at two weeks, CR females exhibited 4.6-fold higher BMAT than CR males after six weeks (Fig. 4A and B). CR significantly reduced Tb.Th, especially in females (Fig. 4C), and modestly influenced trabecular BMD and BV when analysed across all groups (Fig. 4D and E). In contrast, Tb.BV/TV, Tb.Sp, and Tb.N were unaffected by CR (Fig. 4F, G, H). All parameters differed by sex, but no significant time–diet interactions were detected.

Simple linear regression further revealed that BMAT correlated negatively with trabecular BMD, BV, BV/TV, and Tb.N, and positively with Tb.Sp in 6-week CR mice (Fig. 4I, Supplementary Fig. 2C), a pattern largely consistent with the BMAT-bone relationships detected in tibiae (Fig. 2I, Supplementary Fig. 2A). Thus, during CR, BMAT may predict a worsened trabecular bone microarchitecture across multiple bones.

### CR induces greater, duration-dependent reductions in femoral cortical bone in females than in males

We next investigated whether these CR effects extend to femoral cortical bone. CR for six weeks significantly decreased femoral cortical B.Ar, B.Ar/T.Ar, and Cort.Th, with more pronounced diet effects in females than in males (Supplementary Fig. 3A, B, C; AL vs CR heatmaps). Significant location–sex–diet interactions (Supplementary Fig. 3A, B, C) further highlighted spatial differences in these sex-dependent CR responses.

To assess the effect of CR duration, we compared 2-week and 6-week interventions. In males, no significant time–diet or location–time–diet interactions occurred across whole femora (Supplementary Fig. 3D, E, F); however, region-specific analyses identified clear location- and duration-dependent effects, with CR decreasing each parameter more strongly after six vs two weeks. The differences in B.Ar were primarily driven by CR preventing the increases that occurred in AL mice between weeks 2 and 6, whereas the B.Ar/T.Ar differences were mainly due to CR decreasing the absolute values to below those in 2-week or 6-week AL mice.

These cortical changes were greater in females. Here, both B.Ar and Cort.Th increased markedly between weeks 2 and 6 in AL mice; CR prevented these increases, resulting in stronger CR vs AL effects after 6 vs 2 weeks (Supplementary Fig. 3G, H, I). For B.Ar/T.Ar the changes from weeks 2 to 6 were more complex, with reductions observed in the proximal femur under CR and increases in the distal femur under AL. These region-specific changes were accompanied by significant location–time–diet interactions for B.Ar and B.Ar/T.Ar (Supplementary Fig. 3G and H). Collectively, these findings demonstrate sex-dependent and region-specific skeletal responses to CR in the femur, with females exhibiting greater temporal sensitivity than males.

### CR does not increase humeral BMAT but positively impacts humeral trabecular bone in females

We previously demonstrated that BMAT is undetectable in humeri of young mice (27); however, whether it increases during CR is unknown. We found that humeral BMAT remained undetectable even after 30% CR for 6 weeks (data not shown). Thus, to investigate if more extensive CR increases humeral BMAT, we analysed humeri from male mice that underwent graded CR (10, 20, 30, and 40% reduction from AL food intake) for 12 weeks (22). However, humeral BMAT volume did not differ between CR and AL mice, irrespective of the degree of CR (Supplementary Fig. 4).

**Figure 5:**
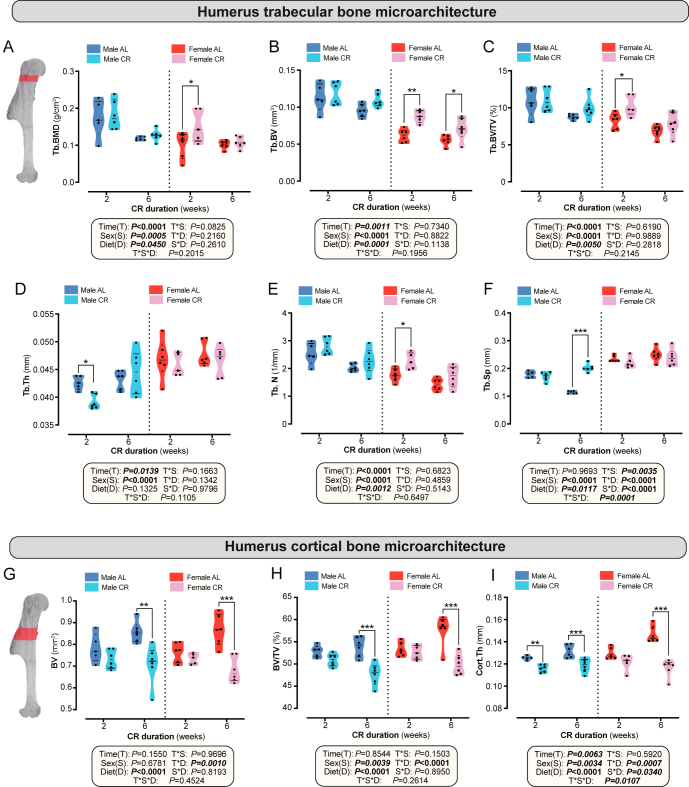
Impact of CR on bone microarchitecture of the humerus. Male and female mice were fed AL or 30% CR diets as described for Fig. 1. (A, B, C, D, E, F) Comparisons of humeral trabecular bone parameters between AL and CR groups in both sexes at week 2 and week 6, including (A) Tb.BMD, (B) Tb.BV, (C) Tb.BV/TV, (D) Tb.Th, (E) Tb.N, and (F) Tb.Sp. (G, H, I) Comparisons of humeral cortical bone parameters between AL and CR groups in both sexes at week 2 and week 6, including (G) BV, (H) BV/TV, and (I) Cort.Th. Data are shown as violin plots (A, B, C, D, E, F, G, H, I) or mean ± SEM (J). Following is the number of mice per group: (A, B, C, D, E, F, G, H, I) male AL, 2 weeks: *n* = 5, 6 weeks: *n* = 6; male CR, 2 weeks: *n* = 6, 6 weeks: *n* = 6; female AL, 2 weeks: *n* = 6, 6 weeks: *n* = 6; and female CR, 2 weeks: *n* = 5, 6 weeks: *n* = 6. Statistical analyses follow the methods detailed in Fig. 2.

In contrast, CR markedly altered humeral bone microarchitecture in a manner distinct from its effects in tibiae and femora. In the metaphysis, CR significantly affected trabecular BMD, BV, BV/TV, Tb.N, and Tb.Sp, with lesser effects on Tb.Th (Fig. 5A, B, C, D, E, F). Among these, only Tb.Sp showed significant time–diet and time–sex–diet interactions (Fig. 5F), suggesting that CR alters Tb.Sp in a sex- and duration-dependent manner. Females generally responded earlier than males, with increasing BMD, BV, BV/TV, and Tb.N after 2-week CR in females only (Fig. 5A, B, C, E). In contrast, males had decreased Tb.Th after 2-week CR and increased Tb.Sp after 6-week CR (Fig. 5D and F). Although CR’s effects were generally stronger in females, significant sex–diet interactions were limited to Tb.Sp (Fig. 5F). Thus, unlike in tibiae and femora, CR can exert positive and sometimes transient effects on humeral trabecular bone: it induces early anabolic or protective effects in females, while males exhibit delayed changes, characterised by increased trabecular spacing.

Regarding cortical bone, CR strongly decreased BV, BV/TV, and Cort.Th, with prominent time–diet interactions (Fig. 5G, H, I). Six weeks of CR decreased BV, BV/TV, and Cort.Th in males and females, whereas two weeks of CR decreased only Cort.Th, and only in males (Fig. 5G, H, I). These findings indicate a sex-specific temporal sensitivity of cortical bone to CR.

### CR reduces caudal vertebral adipocyte size

To determine how CR influences BMAT in axial bones, we used histomorphometry to quantify bone marrow adipocyte sizes in vertebrae of mice after six weeks of AL or CR diet. No adipocytes were detectable in the lumbar vertebrae of AL or CR mice (data not shown). In contrast, adipocytes were abundant in caudal vertebrae, and CR significantly reduced adipocyte sizes in both sexes (Supplementary Fig. 5). Here, males exhibited a more uniform redistribution of adipocyte sizes, whereas females showed a more peaked size distribution.

### CR-induced BMAT expansion is not associated with improved glucose metabolism but correlates with systemic lipid mobilisation

**Figure 6:**
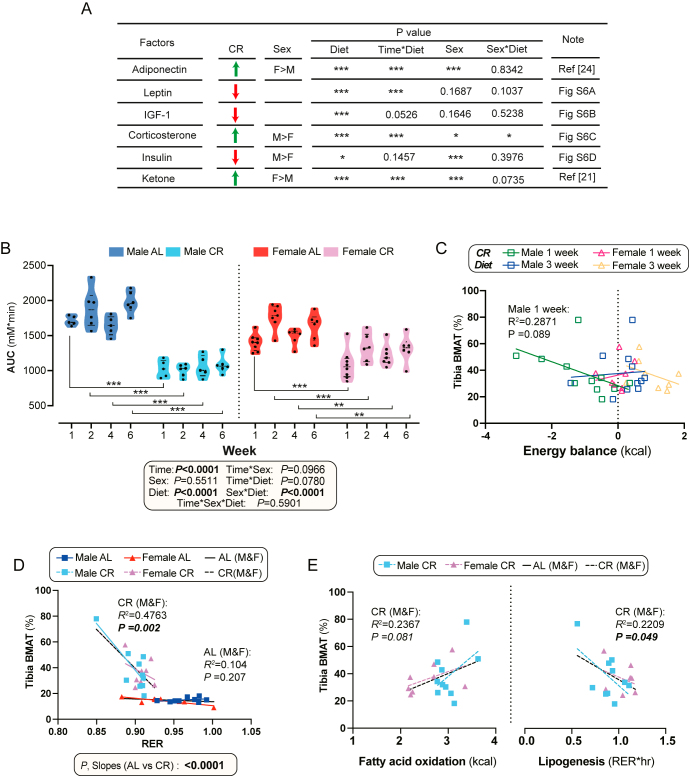
Relationships between BMAT expansion and systemic metabolism under CR. Male and female mice were fed AL or 30% CR diets as described for Fig. 1. (A) Summary table of circulating hormone and metabolite levels in response to CR. (B) Area under the curve (AUC) during oral glucose tolerance tests (OGTTs) determined relative to 0 mmol/L across all time points. (C, D, E) Linear regression used to assess the relationships between tibial BMAT and the indicated metabolically relevant parameters; the diet durations analysed depended upon the experimental groups and time points for which each parameter was measured, as follows: (C) BMAT at week 4 vs energy balance, measured by indirect calorimetry, at week 1 and week 3 of CR; (D) BMAT at week 4 vs respiratory exchange ratio (RER), measured by indirect calorimetry, at week 3 of AL or CR; (E) BMAT at week 4 vs fatty acid oxidation and lipogenesis at week 3 of CR. Data are shown as violin plots (B) or scatter plots (C, D, E) of the following numbers of mice per group: (B) 5–8 mice per group per time point; (C) 1 week male: *n* = 11, female: *n* = 8; 3 weeks male: *n* = 11, female: *n* = 8. (D) Male AL: *n* = 9; male CR: *n* = 10; female AL: *n* = 8; female CR: *n* = 8. (E) male CR: *n* = 10, female CR: *n* = 8. For (A and B), significant effects of diet, time, and sex, as well as their interactions, were assessed using three-way ANOVA. For (B), further differences between AL and CR mice at each time point were assessed by two-way ANOVA with Šidák’s post-tests. Statistical significance is indicated by *P < 0.05, **P < 0.01, ***P < 0.001. For (C, D, E), associations for the indicated groups are shown by *R*^2^ and *P* values within each scatter plot area. For (D), the BMAT-RER relationship did not differ between sexes within each diet; hence, significant AL vs CR differences (*P*, slopes) were assessed for both sexes combined (AL M&F vs CR M&F).

We next investigated the relationships between CR-induced BMAT expansion and the endocrine and metabolic effects of CR. Our goal was to establish the temporal relationships between these phenomena, thereby clarifying whether the endocrine and metabolic responses might be causes or consequences of BMAT expansion. CR significantly increased circulating adiponectin, corticosterone, and ketone concentrations, while leptin, IGF-1, and insulin were decreased (Fig. 6A, Supplementary Fig. 6A, B, C, D) (21, 24). As these circulating factors are closely associated with glucose metabolism (28, 29, 30, 31, 32), we performed OGTTs for each CR duration (Supplementary Fig. 6E, F, G, H). CR enhanced glucose tolerance after only one week, and extending CR to 2–6 weeks had no further effect (Fig. 6B). These CR-induced improvements in glucose tolerance were greater in males than in females, consistent with our previous findings (21). In contrast, BMAT did not significantly increase until after four weeks of CR (Fig. 1B), suggesting that CR-induced improvements in glucose metabolism occur independently of BMAT expansion.

We also found no significant relationships between BMAT expansion and CR’s other metabolic effects, including the degree of weight loss, overall body fat percentage, fold-change in fat mass, or total energy expenditure (Supplementary Fig. 7A, B, C, D, E). Indeed, the extent of CR-induced weight loss was similar at week 1 and week 6 in both males and females – largely because CR mice regain weight from weeks 4 to 6 (21), whereas BMAT levels were significantly higher at week 6 under CR (Supplementary Fig. 7A and B). This suggests that BMAT expansion is not directly related to these metabolic effects of CR. In contrast, during the first week of CR, energy balance tended to inversely correlate with tibial BMAT in males (Fig. 6C) and, across both sexes, RER negatively correlated with tibial BMAT during CR (Fig. 6D). Consistent with this, BMAT tended to positively correlate with fatty acid oxidation and was negatively correlated with lipogenesis during CR (Fig. 6E). The basis for these associations requires further study; however, they support the possibility that enhanced systemic lipid catabolism, accompanied by reduced lipid synthesis, may be a regulatory signal for BMAT expansion during CR.

## Discussion

**Figure 7:**
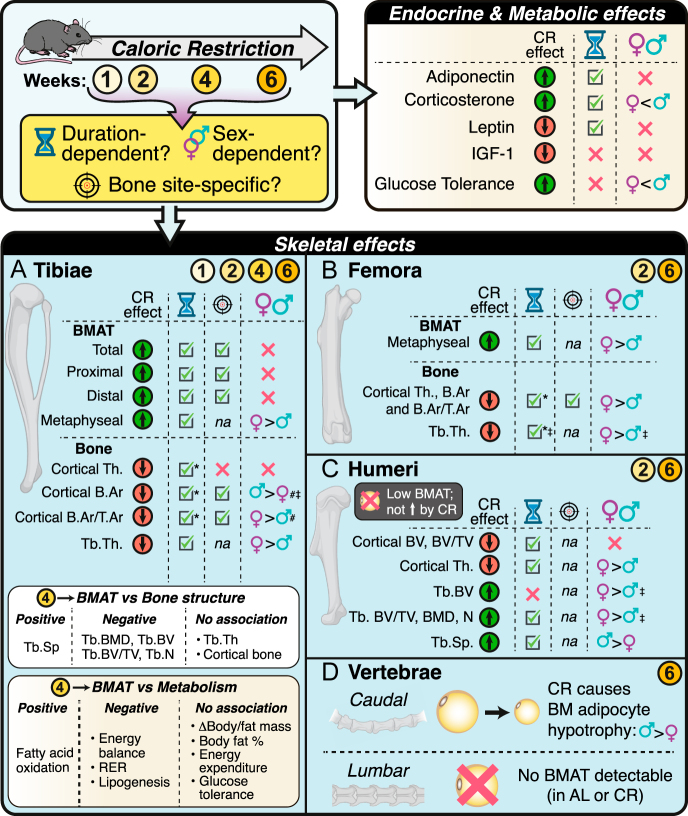
Summary of CR’s duration-, sex-, and site-dependent effects on BMAT, bone, endocrine factors, and metabolic homeostasis. Outcomes are shown only if they are significantly influenced by CR. Numbers in yellow circles indicate the weeks of CR tested for each of the bone analyses. For each outcome (e.g. BMAT, bone, and endocrine/metabolic), duration-, site-, and sex-dependent effects are indicated only if these variables show significant interactions with diet (e.g. sex–diet, time–diet, location–diet) but not if these variables alone affect the outcome. For example, circulating adiponectin is higher in females than in males, regardless of diet, but CR increases adiponectin to a similar extent in both sexes; hence, there is no sex difference in this CR effect. An exception is where variables have more complex influences on the CR response, which are indicated as follows: * = CR has a duration-dependent effect in only one sex or that is stronger in one sex; ^#^ = sex difference in the CR effect is skeletal site dependent; ^‡^ = ANOVA shows no overall sex–diet interaction, but multiple comparisons show stronger CR effects in one sex.

Herein, we demonstrate that CR induces site-specific effects on bone marrow adiposity, with greater BMAT expansion in tibiae than femora; no changes in humeri or lumbar vertebrae; and adipocyte hypertrophy in caudal vertebrae (Fig. 7). These effects are duration- and sex-dependent: longer CR causes greater BMAT expansion, which plateaus after 4 weeks, and whereby females exhibit greater metaphyseal BMAT than males in both tibiae and femora. Spatial analyses along the tibial diaphysis further revealed that BMAT expansion predominates in proximal and distal regions and exhibits further location-dependent sex differences. In tibiae and femora, CR decreases trabecular thickness, especially in females, and decreases cortical bone in a site-specific manner. However, trabecular bone in humeri generally increases with CR, an effect that is also stronger in females (Fig. 7A, B, C). BMAT levels associate more strongly with trabecular than cortical bone parameters, highlighting skeletal subtype-specific relationships. Moreover, CR alters circulating hormones, including adiponectin, leptin, IGF-1, and corticosterone, in a duration- and, sometimes, sex-dependent manner (Fig. 7). Notably, BMAT expansion associates with enhanced systemic fatty acid oxidation and reduced lipogenesis but not with altered glucose tolerance, body mass, total fat mass, or body fat percentage. These findings position BMAT as a metabolically active depot that responds to CR in a duration-, bone-, region-, and sex-specific manner, highlighting potential roles at the interface between systemic energy metabolism and skeletal integrity.

Most previous studies have quantified BMAT primarily through histology of metaphyseal sites (13, 33, 34, 35) or by dividing tibiae into broad anatomical regions (epiphysis, metaphysis, and diaphysis) or proximal and distal compartments (3, 19, 20, 36). Our findings illustrate the spatial and temporal dynamics of BMAT expansion under CR and further provide a novel method for assessing BMAT distribution across appendicular bones. Traditional analysis methods show that females have more BMAT than males in the proximal tibia but no difference in the distal tibia (37); however, we show that AL-fed males have significantly higher distal tibial BMAT than females, a sex difference that is abolished after four weeks of CR. Similar sex differences exist in humans, wherein BMAT exhibits age-, sex-, and site-specific variation, with pronounced changes during menopause in females (5, 38). Indeed, postmenopausal osteoporosis coincides with BMAT accumulation (2), while oestrogen replacement therapy significantly decreases BMAT (39). Human CR studies show that, after ten days of CR, men show greater BMAT accumulation in the L4 vertebrae, whereas women accumulate more in the femoral metaphysis (40). Our findings mirror these patterns, with female mice exhibiting greater BMAT in both tibial and femoral metaphyseal regions compared to males.

We show that tibial cBMAT – often considered to be inert (2, 4), – expands significantly under CR, suggesting that it is more dynamic than previously thought. Intriguingly, from weeks 4 to 6 of CR, females decrease cBMAT at some distal sites (Supplementary Fig. 1E). This raises the possibility that cBMAT may reach a plateau and eventually decrease with prolonged CR, perhaps because of progressive systemic adaptations such as lower energy expenditure (21). Consistent with this plasticity, our recent findings in the UK Biobank show that variation in femoral diaphyseal BMAT – traditionally considered as cBMAT – associates with several human anthropometric traits and diseases (5, 10). Thus, while cBMAT may be less variable than rBMAT, it still shows environmental and pathophysiological plasticity relevant to health and disease.

Our findings shed further light on CR’s skeletal impact. Previous studies have reported inconsistent findings likely due to differences in the skeletal site(s) analysed, age at intervention, sex, and CR duration (13, 33, 34, 41, 42, 43). For example, CR initiated in 8- to 9-week-old mice does not decrease trabecular bone in males and increases trabecular bone in females (23, 33). Life-long CR prevents ageing-associated trabecular bone loss (43), whereas CR initiated in 3-week-old mice, before sexual maturity, decreases femoral trabecular bone (13). Herein, we show that four or six weeks of CR decreases trabecular and cortical bone in tibiae and femora. Notably, within tibiae, BMAT levels during CR associate inversely with trabecular bone but are dissociated from cortical parameters. This highlights site-specific relationships between BMAT and bone loss during CR. Given that trabecular bone is the predominant site of bone marrow stromal cells (BMSCs) (44), this pattern is consistent with a CR-induced shift in BMSC fate towards adipogenesis at the expense of osteogenesis (33). In contrast, CR increases trabecular BMD and BV in humeri, particularly in females, while still impairing cortical bone in both sexes. This is similar to aged mice, in which CR increases trabecular bone but decreases cortical bone within tibiae (36). Notably, unlike tibiae and femora, we show that humeri resist BMAT expansion under CR. Thus, reduced adipogenesis in humeri may allow maintenance of BMSC osteogenesis, thereby helping preserve trabecular structure under CR.

From a clinical perspective, these site-specific alterations in bone remodelling may have important implications for skeletal fragility. Preferential loss of trabecular bone, particularly at BMAT-rich sites, is likely to compromise bone strength and increase fracture risk. In growing individuals, CR-induced accumulation of BMAT and suppression of trabecular bone growth may also limit peak bone mass, with potential long-term consequences for fracture susceptibility. Together, these findings suggest that BMAT expansion may serve as an early indicator of adverse skeletal adaptation to energetic stress.

Consistent with previous studies (17), we show that CR modulates circulating adiponectin, leptin, IGF-1, corticosterone, insulin, and ketone concentrations. We further demonstrate that these changes are duration- and sex-dependent, suggesting differential endocrine adaptations to CR between the sexes and over time. Metabolically, human studies have demonstrated that BMAT has higher basal glucose uptake compared to axial bone and white adipose tissue (27); however, we show that, in mice, improved glucose tolerance under CR does not require BMAT expansion. This extends our previous observations that CR enhances glucose tolerance without altering bone marrow glucose uptake (21). Interestingly, during CR, BMAT becomes a major source of circulating adiponectin (3), a hormone reported to enhance glucose metabolism (45). Our present findings show that circulating adiponectin levels plateau after 3–4 weeks of CR, paralleling the timing of BMAT expansion. This observation further supports BMAT as the primary source of adiponectin and underscores the need to further investigate the roles of adiponectin as a putative mediator of BMAT function under CR (45).

Our study highlights further relationships between BMAT and CR’s systemic metabolic effects. We show that tibial BMAT peaks after four weeks of CR, which is notable because this is also the time at which energy expenditure further declines, weight loss ceases, and CR mice begin to regain weight (21). Thus, BMAT expansion during CR may be associated with overall energy balance. Consistent with this, we show that greater tibial BMAT expansion in males tends to occur in mice that experience a more negative energy balance during the early phase of CR. We propose that greater energy deficit induces adaptations, including suppression of osteogenesis and haematopoiesis, that decrease the demands on BMAT as a local energy source, facilitating its accumulation. Yet counterintuitively, we find BMAT expansion to be significantly associated with a systemic metabolic shift towards increased fatty acid oxidation and reduced lipogenesis. This paradox suggests that systemic lipid mobilization may serve as a signal promoting BMAT accumulation, while locally, BMAT may function as a fuel reservoir to support skeletal and haematopoietic adaptations under CR. Future studies must explore these possibilities.

There are several limitations. First, we may be underpowered to detect BMAT expansion with shorter CR durations. This particularly applies to 2-week CR, for which femoral BMAT tends to increase in females and tibial BMAT tends to increase in both sexes. Despite this limitation, our data will help to inform effect size estimates and power calculations for future studies. Second, plasma hormones were analysed in mice that had not undergone μCT analysis, preventing us from determining the direct relationships between circulating factors and bone phenotypes. Third, the molecular mechanisms underlying CR-induced BMAT expansion remain to be determined. Fourth, femoral μCT analyses were not performed at the 1- or 4-week CR time points, partly because femurs display less BMAT expansion than tibiae, even with 6 weeks of CR. Finally, the effect of CR on bone mass may depend on the age at which CR is initiated. Our AL mice continue to increase body mass (21), and mice on both diets were still undergoing skeletal growth (Supplementary Fig. 1C). Thus, the observed low bone mass may have resulted from CR attenuating bone accrual rather than causing bone loss. If so, our CR regimen may be clinically more relevant to bone loss and BMAT expansion during anorexia nervosa, which typically occurs in adolescents (9), rather than therapeutic CR in older individuals. Future studies should, therefore, further investigate CR’s age-dependent skeletal effects.

In conclusion, our findings establish BMAT as a metabolically responsive depot under CR that exhibits duration-, site-, and sex-specific expansion. This study highlights the dynamic and heterogeneous nature of BMAT across the skeleton and underscores its strong association with trabecular rather than cortical bone remodelling. Furthermore, BMAT accumulation under CR is not directly linked to systemic weight loss or glucose tolerance but instead is associated with negative energy balance, increased fatty acid oxidation, and suppressed lipogenesis. These insights provide a valuable framework for understanding BMAT’s role in skeletal adaptation and systemic energy regulation during nutritional challenges and identify potential mechanisms through which CR might impact skeletal and metabolic health.

## Supplementary materials



## Declaration of interest

The authors declare that there is no conflict of interest that could be perceived as prejudicing the impartiality of the work reported.

## Funding

This work was supported by a PhD scholarship from Tri-Service General Hospital, National Defense Medical Center, Taiwan (to KCC), the UK Medical Research Council (MR/M021394/1 to WPC), the University of Edinburgh (Chancellor’s Fellowship to WPC; PhD Studentship to AL), the Takeda Science Foundation (Fellowship for Young Japanese MDs & PhDs Studying Abroad, to YMI), the Japan Society for the Promotion of Science (JSPS Overseas Research Fellowship, to YMI), the Japan Foundation for Applied Enzymology (to YMI), the British Heart Foundation (PhD Studentship FS/14/60/31283 to RJS and FS/17/62/33477 to BJT), the Wellcome Trust (Grant WT 210752 to RKS; Multi-user equipment grant 223818/Z/21/Z for the Promethion system), and the UK Biotechnology and Biological Sciences Research Council (BBSRC) (Standard grant BB/G009953/1 and China partnering award BB/JO20028/1 to JRS).

## Author contribution statement

Contributions are based on the CRediT (Contributor Roles Taxonomy) and are as follows: KCC, RJS, JRS, and WPC conceptualised the study. KCC, RJS, YMI, and WPC curated the data and performed the formal analysis. KCC, JRS, NMM, RKS, and WPC acquired funding. KCC, RJS, KJS, BJT, AL, AJL, SEM, and WPC conducted the investigation. KCC, RJS, JRS, NMM, and WPC developed the methodology. JRS, NMM, RKS, and WPC administered the project and provided resources. YMI, KJS, JRS, NMM, RKS, and WPC supervised the study. KCC and WPC created the visualisations and prepared the original draft. KCC, KJS, YMI, BJT, SEM, JRS, and WPC reviewed and edited the manuscript.

## Rights retention statement

For the purpose of open access, the authors have applied a Creative Commons Attribution (CC-BY) license to any Author Accepted Manuscript version arising from this submission.
