# Supplementary material for: Caloric restriction exerts site-, sex-, and duration-dependent effects on skeletal structure and bone marrow adiposity
Source: J Endocrinol. 2026 May 11;269(2):e250391. doi: 10.1530/JOE-25-0391 (PMC13188187; doi:10.1530/JOE-25-0391)
Supplement: Supplementary file 1 [file supplementary_materials.pdf]

SUPPLEMENTARY INFORMATION

Caloric restriction exerts site-, sex-, and duration-dependent effects on skeletal structure and bone marrow adiposity

Kuan-Chan Chen, Richard J. Sulston, Karla J. Suchacki, Yoshiko M. Ikushima, Benjamin J. Thomas, Andrea Lovdel, Alex J Lafond, Sharon E. Mitchell, John R. Speakman, Nicholas M. Morton', Robert K. Semple, and William P. Cawthorn

---

This Supplementary Information file contains the following:

- Supplementary Figures 1-7

Figure S1

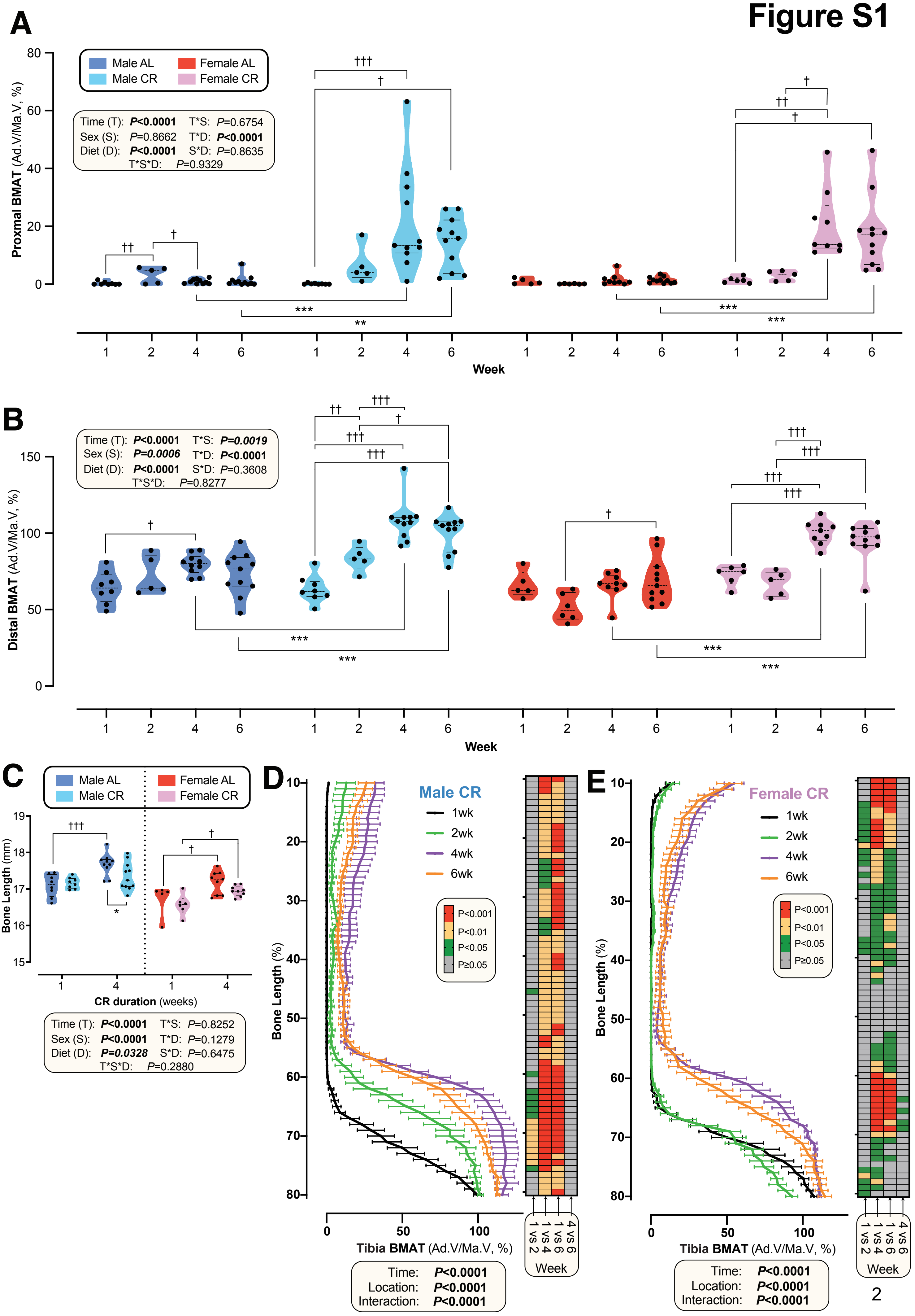

A
Male AL
Male CR
Female AL
Female CR
Time (T): P<0.0001 T*S: P=0.6754
Sex (S): P=0.8662 T*D: P<0.0001
Diet (D): P<0.0001 S*D: P=0.8635
T*S*D: P=0.9329
Proxmal BMAT (Ad.V/Ma.V, %)
Week
B
Time (T): P<0.0001 T*S: P=0.0019
Sex (S): P=0.0006 T*D: P<0.0001
Diet (D): P<0.0001 S*D: P=0.3608
T*S*D: P=0.8277
Distal BMAT (Ad.V/Ma.V, %)
Week
C
Bone Length (mm)
CR duration (weeks)
Time (T): P<0.0001 T*S: P=0.8252
Sex (S): P<0.0001 T*D: P=0.1279
Diet (D): P=0.0328 S*D: P=0.6475
T*S*D: P=0.2880
D
Male CR
1wk
2wk
4wk
6wk
P<0.001
P<0.01
P<0.05
P≥0.05
Bone Length (%)
Tibia BMAT (Ad.V/Ma.V, %)
1 vs 2
1 vs 4
1 vs 6
4 vs 6
Week
Time: P<0.0001
Location: P<0.0001
Interaction: P<0.0001
E
Female CR
1wk
2wk
4wk
6wk
P<0.001
P<0.01
P<0.05
P≥0.05
Bone Length (%)
Tibia BMAT (Ad.V/Ma.V, %)
1 vs 2
1 vs 4
1 vs 6
4 vs 6
Week
Time: P<0.0001
Location: P<0.0001
Interaction: P<0.0001

Supplementary Figure 1 *(related to Figure 1)* – **The impact of CR on tibial BMAT.** Male and female mice were fed either an AL or 30% CR diet starting at 9–10 weeks of age for 1, 2, 4, or 6 weeks. **(A-B)** Quantification of (A) proximal and (B) distal tibial BMAT (%) across timepoints, diets, and sexes. **(C)** Bone length across diets and sexes in week 1 and week 4. **(D–E**) BMAT (%) comparison across timepoints under CR in males (D) and females (E). Data are shown as violin plots (A-C) or mean ± SEM (D-E), and the numbers of mice per group using the same sample sets listed as Figure 1. (A-C) Significant effects of diet, time, sex, and/or location, as well as their interactions, were assessed using three-way ANOVA. Two-way ANOVA and Post hoc Šidák's tests identified (1) differences between AL and CR at each timepoint, and (2) differences among timepoints within each diet group. Significance is indicated by *P<0.05, **P<0.01, ***P<0.001 (AL vs. CR), and †P<0.05, ††P<0.01, †††P<0.001 (within-group timepoint comparisons). (D–E) For heatmap analysis, two-way ANOVA with Fisher's LSD post hoc test was used to compare the effects of CR duration. Statistical significance is color-coded as shown in the legend: red for P < 0.001, yellow for P < 0.01, green for P < 0.05, and gray for P ≥ 0.05.

Figure S2

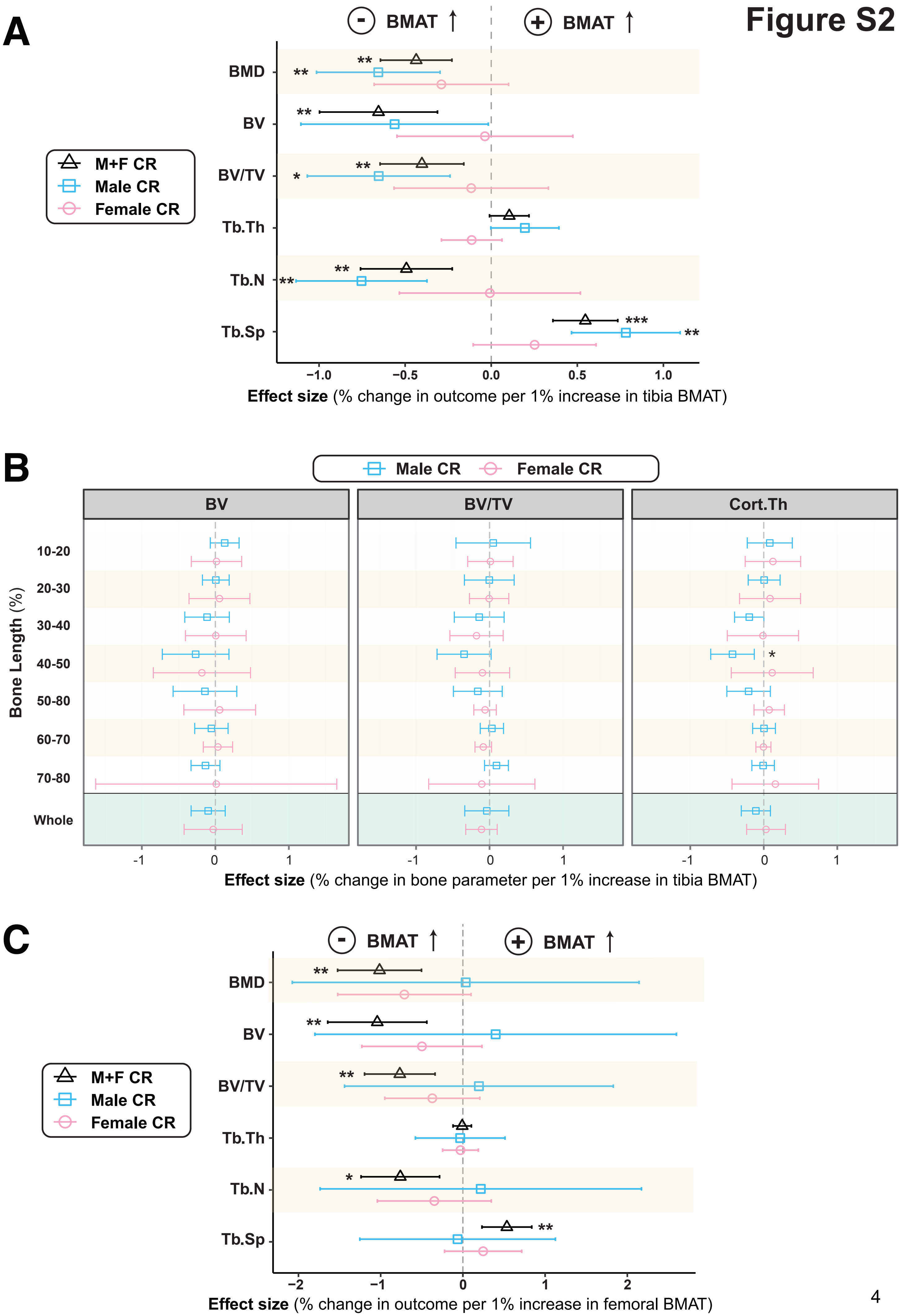
A
BMAT
BMAT
BMD
BV
BV/TV
Tb.Th
Tb.N
Tb.Sp
**
**
**
**
*
**
**
***
**
M+F CR
Male CR
Female CR
−1.0
−0.5
0.0
0.5
1.0
Effect size (% change in outcome per 1% increase in tibia BMAT)
B
Male CR
Female CR
BV
BV/TV
Cort.Th
Bone Length (%)
10-20
20-30
30-40
40-50
50-80
60-70
70-80
Whole
*
-1
0
1
Effect size (% change in bone parameter per 1% increase in tibia BMAT)
C
BMAT
BMAT
BMD
BV
BV/TV
Tb.Th
Tb.N
Tb.Sp
**
**
**
*
**
M+F CR
Male CR
Female CR
−2
−1
0
1
2
Effect size (% change in outcome per 1% increase in femoral BMAT)

Supplementary Figure 2 *(related to Figures 2-4)* – **Associations between BMAT and tibial trabecular and cortical bone, and femoral trabecular bone.**

(A) Linear regression analyses showing the relationships between BMAT and tibial trabecular bone parameters in 4-week CR males and females. **(B)** Linear regression analyses showing the relationships between BMAT and tibial cortical bone parameters within each 10% segment along the tibial diaphysis and across the whole tibia in 4-week CR males and females. **(C)** Linear regression analyses showing the relationships between BMAT and femoral trabecular bone parameters in 6-week CR males and females. Data are shown as forest plots of percentage change in each bone parameter (mean ± 95% confidence interval) per 1% change in BMAT. A significant linear relationship is indicated by * $P < 0.05$,**$P<0.01$, ***$P<0.001$. The numbers of mice per group are as reported for Figure 2 (A), Figure 3 (B) or Figure 4 (C).

Figure S3

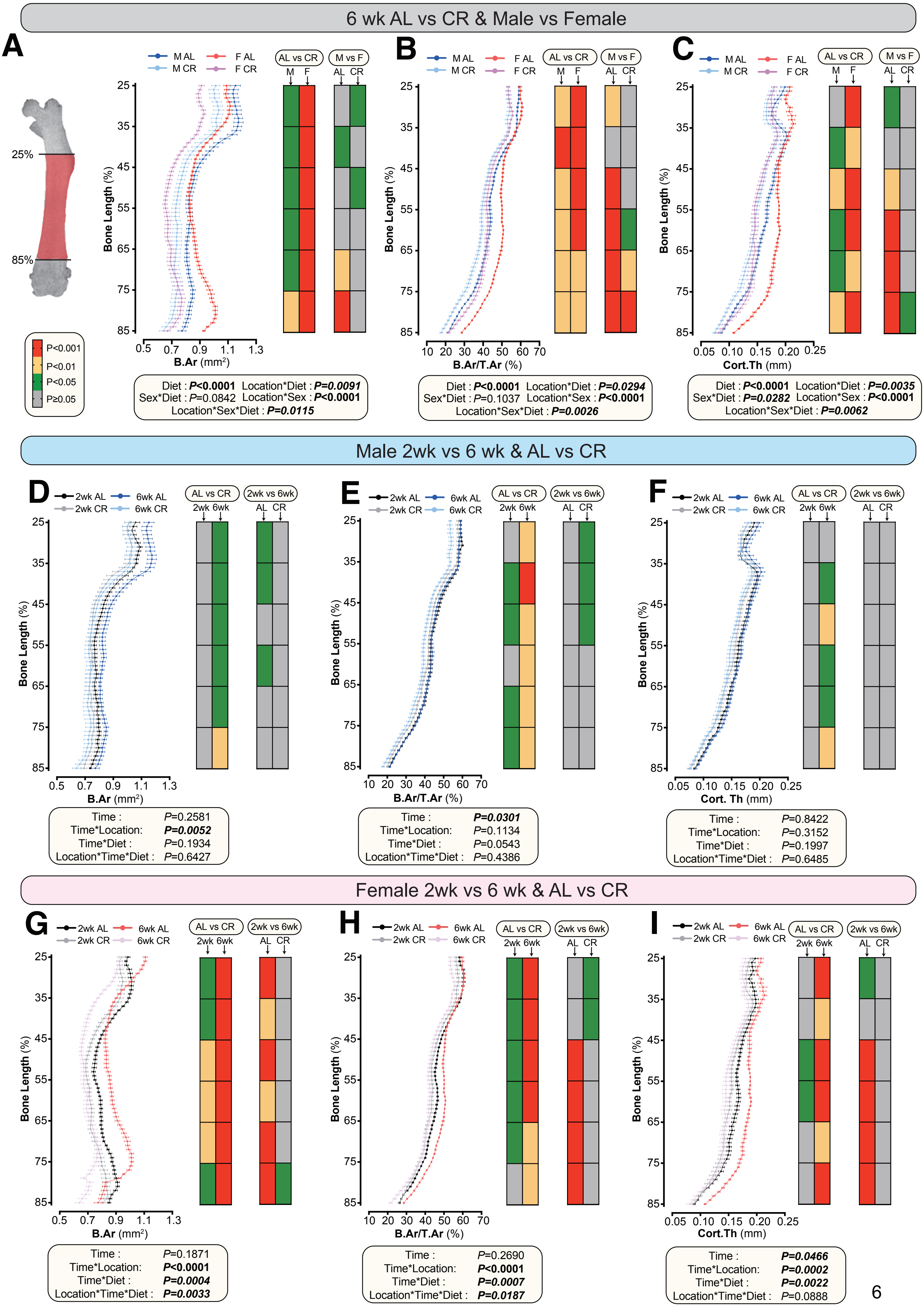

6 wk AL vs CR & Male vs Female
A
B
C
25%
85%
M AL
M CR
F AL
F CR
AL vs CR
M vs F
M
F
AL
CR
Bone Length (%)
B.Ar (mm²)
B.Ar/T.Ar (%)
Cort.Th (mm)
P<0.001
P<0.01
P<0.05
P≥0.05
Diet : P<0.0001 Location*Diet : P=0.0091
Sex*Diet : P=0.0842 Location*Sex : P<0.0001
Location*Sex*Diet : P=0.0115
Diet : P<0.0001 Location*Diet : P=0.0294
Sex*Diet : P=0.1037 Location*Sex : P<0.0001
Location*Sex*Diet : P=0.0026
Diet : P<0.0001 Location*Diet : P=0.0035
Sex*Diet : P=0.0282 Location*Sex : P<0.0001
Location*Sex*Diet : P=0.0062
Male 2wk vs 6 wk & AL vs CR
D
E
F
2wk AL
2wk CR
6wk AL
6wk CR
2wk vs 6wk
2wk
6wk
Cort. Th (mm)
Time : P=0.2581
Time*Location: P=0.0052
Time*Diet : P=0.1934
Location*Time*Diet : P=0.6427
Time : P=0.0301
Time*Location: P=0.1134
Time*Diet : P=0.0543
Location*Time*Diet : P=0.4386
Time : P=0.8422
Time*Location: P=0.3152
Time*Diet : P=0.1997
Location*Time*Diet : P=0.6485
Female 2wk vs 6 wk & AL vs CR
G
H
I
Time : P=0.1871
Time*Location: P<0.0001
Time*Diet : P=0.0004
Location*Time*Diet : P=0.0033
Time : P=0.2690
Time*Location: P<0.0001
Time*Diet : P=0.0007
Location*Time*Diet : P=0.0187
Time : P=0.0466
Time*Location: P=0.0002
Time*Diet : P=0.0022
Location*Time*Diet : P=0.0888

Supplementary Figure 3. Impact of CR on cortical bone microarchitecture in the femur. Male and female mice were fed AL or 30% CR diets as described for Figure 1. **(A–C)** Comparisons of femoral cortical bone parameters between AL and CR groups after 4 weeks of of AL or CR feeding, across 25–85% of femoral length in both sexes. (A) B.Ar, (B) B.Ar/T.Ar, and (C) Cort.Th. **(D–I)** Comparisons of femoral bone parameters between 1-week and 4-week AL and CR groups in males (D–F) and females (G–I) across 25–85% of femoral length: (D,G) B.Ar, (E,H) B.Ar/T.Ar, and (F,I) Cort.Th. Data are presented as mean ± SEM (A–I). Sample sizes are as described in Figure 4, and statistical analyses follow the methods detailed in Figure 3.

Figure S4

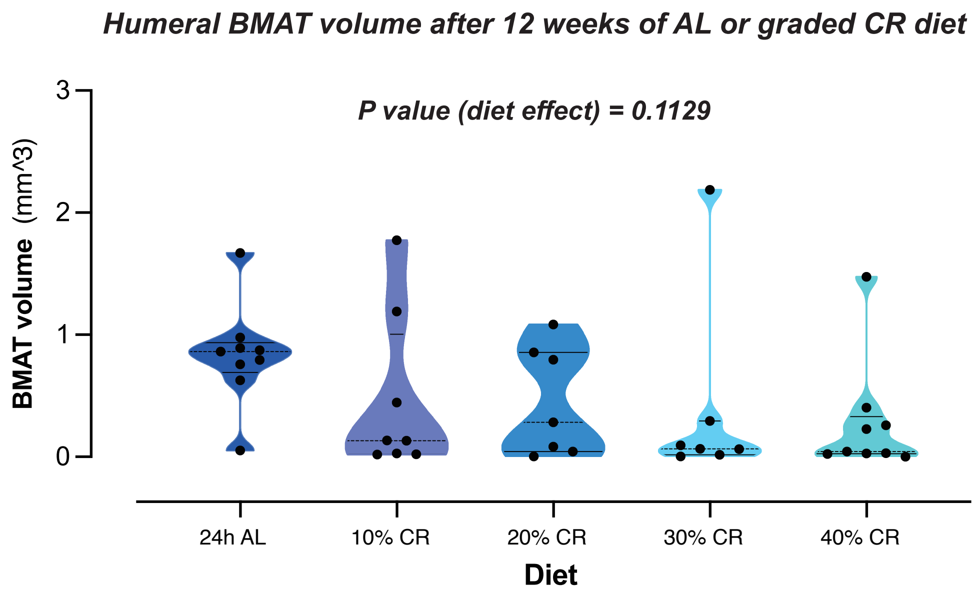

Supplementary Figure 4 *(related to Figure 5)* – **Quantification of humeral BMAT volume from 12 weeks of graded CR in male mice.**

Twenty-week-old male C57BL/6 mice were fed AL or increasing extents of CR (10-40%) for 12 weeks. BMAT volume was then determined from µCT analysis of osmium-tetroxide-stained humeri. Data are shown as violin plots of the following numbers of mice per group: AL, n=9; 10% CR, n=8; 20% CR, n=7; 30% CR, n=7; 40% CR, n=9. Significant effects of diet were assessed using the Kruskal–Wallis test, followed by post hoc Dunn's tests to identify differences between AL and CR groups; no significant AL vs CR differences were identified.

Figure S5

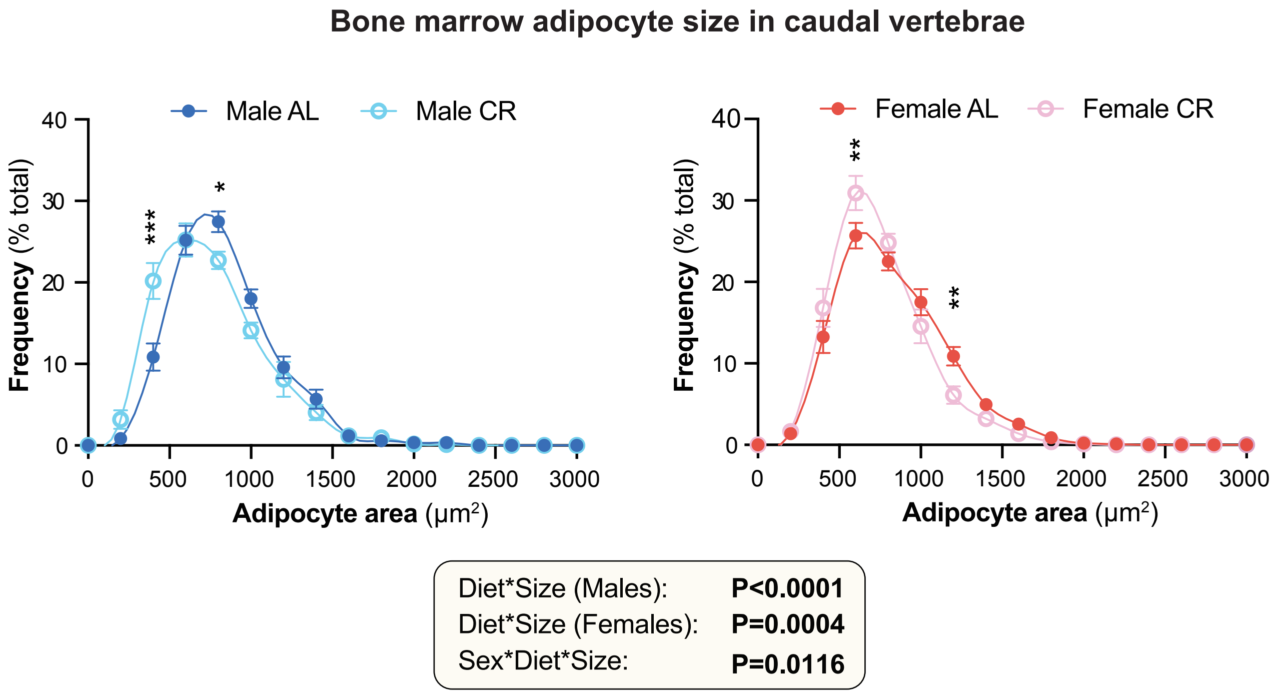

Supplementary Figure 5 – Quantification of adipocyte size of caudal vertebrae after 6 weeks of AL or CR diet.

Histomorphometric analysis of vertebral adipocyte area between AL and CR groups in males (left) and females (right) at week 6. Significant effects of diet, sex, and their impact on the distribution of adipocyte sizes were determined by 3-way ANOVA. Data are presented as mean ± SEM of the following number per group: Male AL n=4; Male CR n=6; Female AL n=6; Female CR n=6.

Figure S6

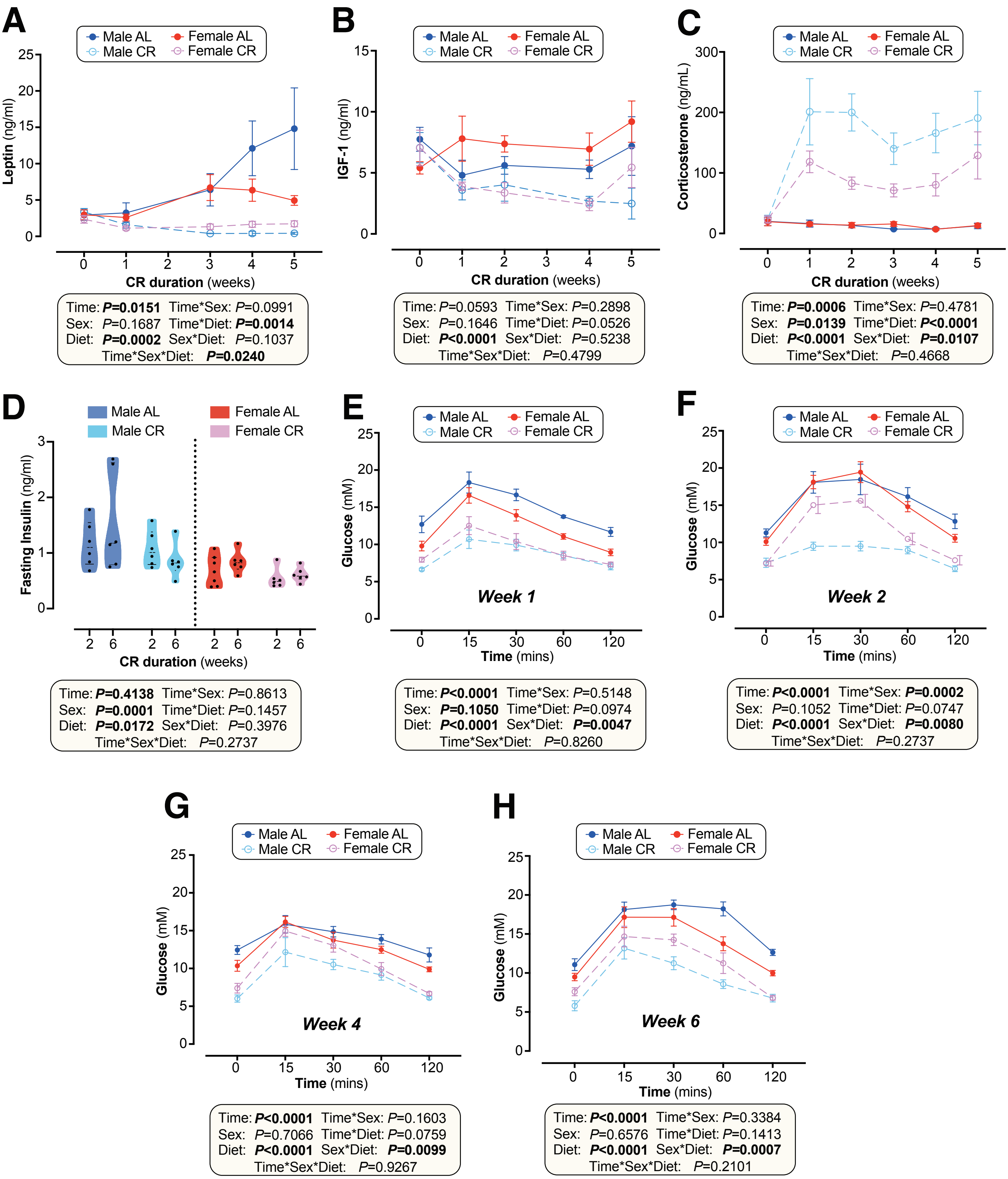
A
Male AL
Male CR
Female AL
Female CR
Leptin (ng/ml)
CR duration (weeks)
Time: P=0.0151 Time*Sex: P=0.0991
Sex: P=0.1687 Time*Diet: P=0.0014
Diet: P=0.0002 Sex*Diet: P=0.1037
Time*Sex*Diet: P=0.0240
B
IGF-1 (ng/ml)
CR duration (weeks)
Time: P=0.0593 Time*Sex: P=0.2898
Sex: P=0.1646 Time*Diet: P=0.0526
Diet: P<0.0001 Sex*Diet: P=0.5238
Time*Sex*Diet: P=0.4799
C
Corticosterone (ng/mL)
CR duration (weeks)
Time: P=0.0006 Time*Sex: P=0.4781
Sex: P=0.0139 Time*Diet: P<0.0001
Diet: P<0.0001 Sex*Diet: P=0.0107
Time*Sex*Diet: P=0.4668
D
Fasting Insulin (ng/ml)
CR duration (weeks)
Time: P=0.4138 Time*Sex: P=0.8613
Sex: P=0.0001 Time*Diet: P=0.1457
Diet: P=0.0172 Sex*Diet: P=0.3976
Time*Sex*Diet: P=0.2737
E
Glucose (mM)
Week 1
Time (mins)
Time: P<0.0001 Time*Sex: P=0.5148
Sex: P=0.1050 Time*Diet: P=0.0974
Diet: P<0.0001 Sex*Diet: P=0.0047
Time*Sex*Diet: P=0.8260
F
Glucose (mM)
Week 2
Time (mins)
Time: P<0.0001 Time*Sex: P=0.0002
Sex: P=0.1052 Time*Diet: P=0.0747
Diet: P<0.0001 Sex*Diet: P=0.0080
Time*Sex*Diet: P=0.2737
G
Glucose (mM)
Week 4
Time (mins)
Time: P<0.0001 Time*Sex: P=0.1603
Sex: P=0.7066 Time*Diet: P=0.0759
Diet: P<0.0001 Sex*Diet: P=0.0099
Time*Sex*Diet: P=0.9267
H
Glucose (mM)
Week 6
Time (mins)
Time: P<0.0001 Time*Sex: P=0.3384
Sex: P=0.6576 Time*Diet: P=0.1413
Diet: P<0.0001 Sex*Diet: P=0.0007
Time*Sex*Diet: P=0.2101

Supplementary Figure 6 *(related to Figure 6)* – **Duration-dependent effects of CR on endocrine factors and glucose tolerance.**

Male and female mice were fed either an AL or 30% CR diet starting at 9–10 weeks of age for 1, 2, 4, or 6 weeks. **(A–C)** Plasma concentrations of (A) leptin, (B) IGF-1, and (C) corticosterone were measured weekly using ELISA from tail vein blood. **(D)** Plasma insulin levels at time 0 of the OGTT were measured at each timepoint using ELISA. **(E–H)** Blood glucose levels measured during the OGTT at week 1 (E), week 2 (F), week 4 (G), and week 6 (H) across both diets and sexes. Data are shown as mean ± SEM for each timepoint (A-C, E-H) or as violin plots (D) of the following numbers of mice: (A) n = 5 per group (except female groups at weeks 0–1: n = 9–10; male CR: week 3 = 4, week 4 = 3, week 5 = 4); (B) n = 4–8 per group per timepoint (except female CR week 1: n = 2; male CR week 4: n = 3; week 5: n = 2); (C) 11-15 mice per group per timepoint; (D) 6-7 mice per group per timepoint. (E-H) 5-8 mice per group per timepoint. Significant effects of diet, time, and sex, as well as their interactions, were assessed using a mixed-effects model (A-D) or three-way ANOVA (E-H) and are presented as for previous figures.

Figure S7

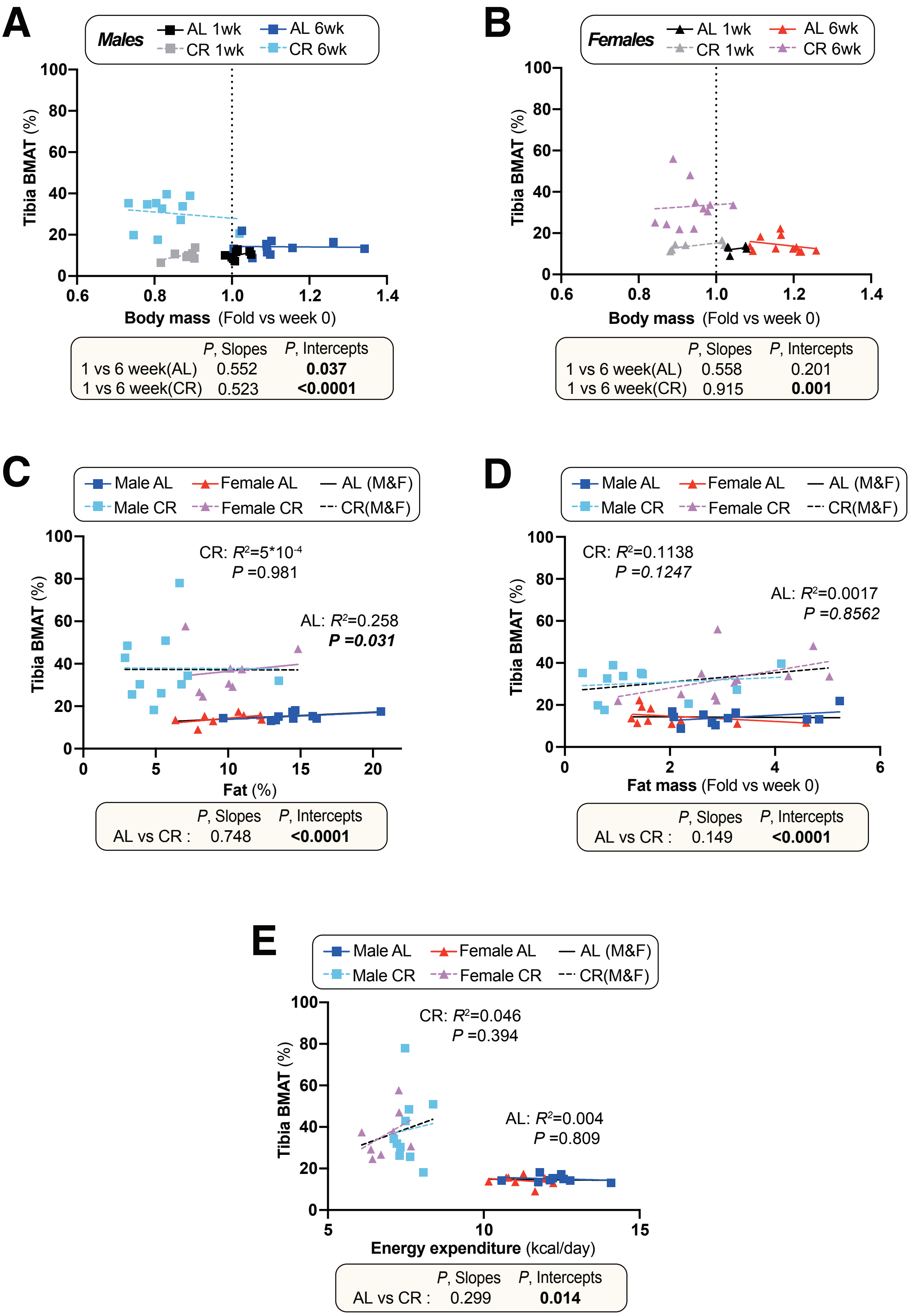
A
Males
AL 1wk
AL 6wk
CR 1wk
CR 6wk
Tibia BMAT (%)
Body mass (Fold vs week 0)
P, Slopes P, Intercepts
1 vs 6 week(AL) 0.552 0.037
1 vs 6 week(CR) 0.523 <0.0001
B
Females
AL 1wk
AL 6wk
CR 1wk
CR 6wk
Tibia BMAT (%)
Body mass (Fold vs week 0)
P, Slopes P, Intercepts
1 vs 6 week(AL) 0.558 0.201
1 vs 6 week(CR) 0.915 0.001
C
Male AL
Female AL
AL (M&F)
Male CR
Female CR
CR(M&F)
CR: R²=5*10⁻⁴
P =0.981
AL: R²=0.258
P =0.031
Tibia BMAT (%)
Fat (%)
P, Slopes P, Intercepts
AL vs CR : 0.748 <0.0001
D
Male AL
Female AL
AL (M&F)
Male CR
Female CR
CR(M&F)
CR: R²=0.1138
P =0.1247
AL: R²=0.0017
P =0.8562
Tibia BMAT (%)
Fat mass (Fold vs week 0)
P, Slopes P, Intercepts
AL vs CR : 0.149 <0.0001
E
Male AL
Female AL
AL (M&F)
Male CR
Female CR
CR(M&F)
CR: R²=0.046
P =0.394
AL: R²=0.004
P =0.809
Tibia BMAT (%)
Energy expenditure (kcal/day)
P, Slopes P, Intercepts
AL vs CR : 0.299 0.014

Supplementary Figure 7 *(related to Figure 6)* **– Relationships between BMAT expansion and body mass, fat mass or energy expenditure in AL and CR mice. (A-E)** Linear regression was used to assess the relationships between tibial BMAT and the indicated metabolically relevant parameters; the diet durations analysed depended upon the experimental groups and timepoints for which each parameter was measured. (A-B) BMAT vs body mass for AL and CR males (A) and females (B) at week 1 and week 6. (C) BMAT vs body fat percentage, as assessed by TD-NMR after 4 weeks of AL or CR diet. (D) BMAT vs fat mass fold change between week 0 and week 6 of AL or CR diet. (E) BMAT at week 4 vs energy expenditure, measured by indirect calorimetry, at week 3. Data are shown as scatter plots of the following numbers of mice per group: (A) Male AL, n=8 (1 wk) or 11 (6 wk); Male CR, n=8 (1 wk) or 11 (6 wk). (B) Female AL, n=5 (1 wk) or 11 (6 wk); Female CR, n=6 (1 wk) or 11 (6 wk). (C) Male AL, n=10; Male CR, n=11; Female, AL n=8; Female CR, n=8. (D) Male AL, n=11; Male CR, n=11; Female AL, n=11; Female CR, n=11. (E) Male AL, n=9; Male CR, n=10; Female, AL n=8; Female CR, n=8. For each comparison, *P* values for significant differences in the regression line slopes and intercepts between 1-week vs 6-week (A-B) and AL vs CR groups(C-E) are shown beneath each graph. For (C-E), none of the relationships differed between sexes within each diet; thus, AL vs CR differences were assessed by combining the sexes within each diet group (AL M&F vs CR M&F). None of the slopes was significantly non-zero, as indicated by the P values within the scatter plot boundaries.
